# *Cryptosporidium sciurinum* n. sp. (Apicomplexa: Cryptosporidiidae) in Eurasian Red Squirrels (*Sciurus vulgaris*)

**DOI:** 10.3390/microorganisms9102050

**Published:** 2021-09-28

**Authors:** Jitka Prediger, Jana Ježková, Nikola Holubová, Bohumil Sak, Roman Konečný, Michael Rost, John McEvoy, Dušan Rajský, Martin Kváč

**Affiliations:** 1 Faculty of Agriculture, University of South Bohemia in České Budějovice, Studentská 1668, 370 05 České Budějovice, Czech Republic; jitka.prediger@gmail.com (J.P.); jezkja@seznam.cz (J.J.); nikoleto@seznam.cz (N.H.); konecnyroman@centrum.cz (R.K.); rost@jcu.cz (M.R.); 2 Institute of Parasitology, Biology Centre of the Czech Academy of Sciences, Branišovská 31, 370 05 České Budějovice, Czech Republic; casio@paru.cas.cz; 3 Microbiological Sciences Department, North Dakota State University, 1523 Centennial Blvd, Van Es Hall, Fargo, ND 58102, USA; john.mcevoy@ndsu.edu; 4 Faculty of Forestry, Technical University in Zvolen, T.G. Masaryka 24, 960 01 Zvolen, Slovakia; dusan.rajsky@gmail.com

**Keywords:** occurrence, biology, course of infection, infectivity, oocyst size, phylogeny, *Cryptosporidium* sp. ferret genotype

## Abstract

*Cryptosporidium* spp. are common protozoan pathogens in mammals. The diversity and biology of *Cryptosporidium* in tree squirrels are not well studied. A total of 258 Eurasian red squirrels (*Sciurus vulgaris*) from 25 and 15 locations in the Czech Republic and Slovakia, respectively, were examined for *Cryptosporidium* spp. oocysts and specific DNA at the *SSU*, actin, *HSP*70, *TRAP-C1*, *COWP*, and *gp60* loci. Out of 26 positive animals, only juveniles (9/12) were microscopically positive (18,000 to 72,000 OPG), and molecular analyses revealed the presence of *Cryptosporidium* sp. ferret genotype in all specimens. Oocysts obtained from naturally-infected squirrels measured 5.54–5.22 μm and were not infectious for laboratory mice (BALB/c and SCID), Mongolian gerbils, Guinea pigs, Southern multimammate mice, chickens, or budgerigars. None of naturally infected squirrels showed clinical signs of disease. The frequency of occurrence of the ferret genotype in squirrels did not vary statistically based on host age, gender or country of capture. Phylogenetic analysis of sequences from six loci revealed that *Cryptosporidium* sp. ferret genotype is genetically distinct from the currently accepted *Cryptosporidium* species. Morphological and biological data from this and previous studies support the establishment of *Cryptosporidium* sp. ferret genotype as a new species, *Cryptosporidium* *sciurinum* n. sp.

## 1. Introduction

*Cryptosporidium* is a genus of single-cell protist parasites that infect the gastrointestinal, respiratory, and/or the urogenital tract of most vertebrates, including humans, causing the disease cryptosporidiosis [1]. The course of infection and severity of the disease depends on a host factors such as age, competence and maturity of the immune system, condition, the presence of secondary infections, and on the characteristics of the *Cryptosporidium* species or genotype [2]. Most of the species validly described so far (48) are narrowly host specific, and only a small number, including *C. parvum*, *C. ubiquitum*, and *C. baileyi*, have a broad host range [3,4]. In addition to the validly described species, dozens of genotypes have been described that lack sufficient data to justify a species designation. Genotypes are typically named for the host from which the novel *Cryptosporidium* DNA sequence is first identified. For example, *C. suis* was originally described as the *Cryptosporidium* pig genotype because it was first identified in a pig [5]. New genotypes are most frequently described from partial sequences of the small subunit rRNA gene. The squirrel, family *Sciuridae*, is one of the most diverse and widely distributed families of mammals, and they host several species and genotypes of *Cryptosporidium*, some of which are infectious to humans and can cause severe and life-threatening diarrhoea [6]. 

Tree squirrels diverged from ground squirrels about 30 million years ago, and this polyphyletic tribe comprises hundreds of extant species. *Cryptosporidium* was first reported in a tree squirrel in 1982, when Sundberg et al. [7] isolated oocysts from an eastern grey squirrel (*Sciurus carolinensis*). Current [8] subsequently reported *Cryptosporidium* oocysts in a fox squirrel (*Sciurus niger*). Both authors identified the isolates as *C. parvum*, but this was likely incorrect, based on our present knowledge. We now know that several distinct *Cryptosporidium* species and genotypes with overlapping oocyst sizes infect tree squirrels. Four species and five genotypes of *Cryptosporidium* have been described in tree squirrels inhabiting North America: *C. ubiquitum*, *Cryptosporidium* sp. chipmunk genotype I, deer mouse genotype III, and skunk genotype in the American red squirrel (*Tamiasciurus hudsonicus*); *C. baileyi*, *C. muris*, *C. ubiquitum*, *Cryptosporidium* sp. chipmunk genotype I, deer mouse genotype III, and skunk genotype in the Eastern grey squirrel (*Sciurus carolinensis*); *C. ubiquitum* in the Fox squirrel (*Sciurus niger*) [7,9,10,11,12]. Fewer studies have reported on *Cryptosporidium* in tree squirrels outside of North America. In China, *C. parvum*, *C. wrairi*, and *Cryptosporidium* sp. rat genotype II have been detected in wild Pallas’s squirrels (*Callosciurus erythraeus*), and *C. parvum*, *C. ratti*, *Cryptosporidium* sp. rat genotype II, ferret genotype, and chipmunk genotype III have been reported in Eurasian red squirrels [13,14,15]. In Europe, native Eurasian red squirrels are almost exclusively infected with *Cryptosporidium* sp. ferret genotype [16,17], although other *Cryptosporidium* species and genotypes, such as *C. ubiquitum*, *Cryptosporidium* sp. skunk genotype, and chipmunk genotype I, have been reported in areas where the Eurasian red squirrel is threatened by invasive, non-native species from Asia and North America [16,17]. It can be concluded from the literature that Eurasian red squirrels primarily host *Cryptosporidium* sp. ferret genotype, whereas the *C. ubiquitum* and *Cryptosporidium* skunk genotypes are most common in North American squirrels [7,9,10,11,12,13,14,15,16,17].

This study aimed to describe the biological, morphological, and genetic characteristics of *Cryptosporidium* sp. ferret genotype from Eurasian red squirrels in Central Europe. Our data show that the ferret genotype is the major *Cryptosporidium* species infecting Eurasian red squirrels in this region, and it is genetically and biologically distinct from valid *Cryptosporidium* species. We therefore propose that it be named *Cryptosporidium sciurinum* n. sp.

## 2. Materials and Methods

### 2.1. Ethics Statement 

Traps were checked at least twice per day and handling time was minimized to reduce animal stress; all applicable international, national, and institutional guidelines for the care and use of animals were followed. Permits for trapping and handling squirrels and for the transmission study complied with the laws of the Czech Republic (Act No. 246/1992 Coll., on the protection of animals against cruelty). The study design was approved by the ethical committees at the Biology Centre of CAS, the State Veterinary Administration, and the Central Commission for Animal Welfare under protocol Nos. MZP/2019/603/1411 and 35/2018.

### 2.2. Trapping and Specimen Collection

**Figure 1:**
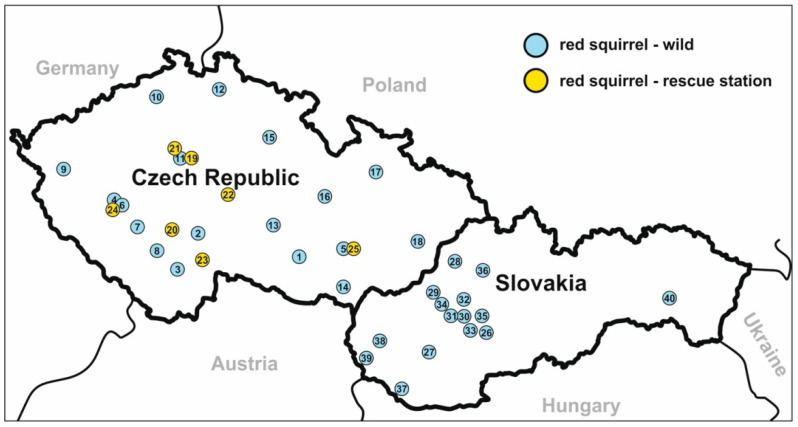
Sampling localities in the Czech Republic and Slovakia: For each site, the number indicates the name of locations (**1**) Třebíč; (**2**) Brandlín; (**3**) České Budějovice; (**4**) Plzeň; (**5**) Brno; (**6**) Starý Plzenec; (**7**) Chocenice; (**8**) Strakonice; (**9**) Mariánské Lázně; (**10**) Ústí nad Labem; (**11**) Praha; (**12**) Liberec; (**13**) Jihlava; (**14**) Břeclav; (**15**) Jaroměř; (**16**) Svitavy; (**17**) Bruntál; (**18**) Valašské Meziříčí; (**19**) Jinonice; (**20**) Makov; (**21**) Praha; (**22)** Vlašim; (**23**) Třeboň; (**24**) Plzeň; (**25**) Brno; (**26**) Zvolen; (**27**) Nitra; (**28**) Považská Bystrica; (**29**) Trenčín; (**30)** Handlová; (**31**) Prievidza; (**32**) Turčianské Teplice; (**33**) Žiar nad Hronom; (**34**) Bánovce nad Bebravou; (**35**) Kremnica; (**36**) Martin; (**37**) Gabčíkovo; (**38**) Pezinok; (**39**) Bratislava; (**40**) Košice.

Faecal samples were collected between June 2019 and June 2021 from Eurasian red squirrels at 25 locations, including seven rescue stations in the Czech Republic and 15 locations in Slovakia (Figure 1). A total of 265 faecal samples were obtained from 258 Eurasian red squirrels. Of these, 147 samples were from squirrels trapped in the wild and 111 were from animals housed at rescue stations. Two squirrels kept at a rescue station (nos. 45901 and 51489) were screened repeatedly. Wild squirrels were live-captured in a Sherman box baited with a mixture of nuts and seeds. Each trapped squirrel was placed into a wire-mesh handling cone to minimize stress during handling and the sex, age, and body condition were recorded following [18]. The presence of any faecal material on the fur around the rectum was also recorded. After release of the trapped animal, faecal samples were collected from traps and individually placed in a sterile plastic tube, which was stored at 4–8 °C until subsequent processing. The consistency of faecal samples was recorded at the time of collection. A faecal smear was prepared from each sample, stained with aniline-carbol-methyl violet (ACMV) [19], and examined for the presence of *Cryptosporidium* spp. oocysts. Oocysts were quantified and the infection intensity was estimated using the method described by Kváč et al. [20]. Briefly, the slide was weighed to the nearest 0.001 g before and after preparation of the smear to determine the mass of faecal material added to the slide. After ACMV staining, all oocysts on the slide were counted and the number of oocysts per gram of faeces was calculated. Oocysts were enumerated from triplicate smears prepared from each sample. All samples were subsequently screened in triplicate for the presence of *Cryptosporidium*-specific DNA by PCR/sequencing of a fragment of the small subunit rRNA gene (SSU). If *Cryptosporidium*-specific DNA was detected, genotyping at other loci was performed as described below.

### 2.3. Source of Oocysts of Cryptosporidium *sp.* Ferret Genotype

An isolate of *Cryptosporidium* sp. ferret genotype was obtained from a juvenile Eurasian red squirrel at a rescue station (isolate 45901, locality no. 23). Faecal samples from this squirrel were individually collected into sterile 50 mL vials with a few drops of dH_2_O and stored at 4–8 °C. Oocysts were purified using caesium chloride gradient centrifugation [21] and used for morphometry and transmission studies (described below). Oocyst viability was examined following propidium iodide (PI) staining by a modified assay of Sauch et al. [22]. The oocysts were stored in PBS at 4–8 °C for a maximum of 4 weeks.

### 2.4. Molecular Characterization

Total genomic DNA (gDNA) was extracted from 5000 purified oocysts, 200 mg of faeces or 200 mg of tissue (see Clinical and Pathomorphological Examinations) by bead disruption (all sample types) for 60 s at 5.5 m/s using 0.5 mm glass beads in a FastPrep^®^24 Instrument (MP Biomedicals, CA, USA) followed by isolation/purification using Exgene^TM^ Stool DNA mini (GeneAll Biotechnology Co. Ltd., Seoul, Korea) or DNeasy Blood & Tissue Kit (Qiagen, Hilden, Germany) in accordance with the manufacturer’s instructions. Purified DNA was stored at −20 °C prior to amplification by PCR. Nested-PCR protocols were used to amplify partial sequences of genes encoding *SSU*, actin, the 70 kDa heat shock protein (*HSP70*), the thrombospondin-related adhesive protein of *Cryptosporidium*-1 (*TRAP-C1*), *Cryptosporidium* oocyst wall protein (*COWP*), and the 60 kDa glycoprotein (*gp60*) using previously published protocols and primers [23,24,25,26,27,28,29]. Negative (molecular grade water) and positive controls (DNA of *C. tyzzeri* subtype family XIa) were included in each PCR amplification.

### 2.5. Sequence and Phylogenetic Analysis

Secondary PCR products were purified using Gen Elute Gel Extraction Kit (Sigma, St. Louis, MO, USA) and sequenced in both directions using an ABI Prism™ Dye Terminator Cycle Sequencing kit (Applied Biosystems, Foster City, CA, USA) using secondary PCR primers according to the manufacturer’s instructions in a commercial laboratory (SEQme, Dobříš, Czech Republic). Sanger sequencing chromatogram files were edited using the ChromasPro 2.1.8 software (Technelysium, Pty, Ltd., South Brisbane, Australia), the obtained nucleotide sequences of each gene were aligned with each other and with reference sequences from GenBank (https://www.ncbi.nlm.nih.gov, accessed on 9 August 2021) using MAFFT version 7 online server (http://mafft.cbrc.jp/alignment/software/, accessed on 9 August 2021) and manually edited and trimmed using BioEdit v.7.0.5 [30]. Maximum likelihood (ML) and Neighbor-joining (NJ) trees were constructed using the Molecular Evolutionary Genetics Analysis (MEGAX) software after computing the most appropriate evolutionary models and values of all parameters for each model. Bootstrap support for branching was based on 1000 replications. Estimates of pairwise distances between species as the number of base substitutions per site from between sequences were calculated in MEGAX. Sequences have been deposited in GenBank under the accession numbers MZ726453–MZ726454 (*SSU*), MZ772035–MZ772036 (actin), MZ772046–MZ772047 (*HSP70*), MZ772037–MZ772038 (*TRAP-C1*), MZ772044–MZ772045 (*COWP*), and MZ772039–MZ772042 (*gp60*).

### 2.6. Oocyst Morphometry

The length and width of *Cryptosporidium* sp. ferret genotype oocysts (*n* = 50) was determined using a digital analysis of images (Olympus cellSens Entry 2.1 software, Olympus Corporation, Shinjuku, Tokyo, Japan) collected using an Olympus Digital Colour Camera DP73 (Olympus), using differential interference contrast (DIC) microscopy at 1000× magnification (Olympus IX70, Tokyo, Japan). These measurements were used to calculate the mean length, width, and the length-to-width ratio. Oocyst size was measured using the same microscope and by the same person. Faecal smears with oocysts of *Cryptosporidium* sp. ferret genotype were stained by modified ACMV, Ziehl–Neelsen (ZN; [31]) and labelled with a Cy3-labelled mouse monoclonal antibody targeting the Cryptosporidium oocyst outer wall antigenic sites (A400Cy2R-20X, Crypt-a-Glo, Waterborne, Inc., New Orleans, LA, USA) and with genus-specific, FITC-conjugated antibodies (IFA; *Cryptosporidium* IF Test, Crypto cel, Cellabs Pty Ltd., Brookvale, Australia). Type microphotographs of oocysts were taken from each staining/labelling.

### 2.7. Transmission Studies

All animals used in transmission studies were screened every day for the presence of oocysts of *Cryptosporidium* spp. and specific DNA (*SSU*) a week prior to transmission studies. Five seven-day-and-eight-week-old SCID (strain C.B-17) and BALB/c mice (*Mus musculus*), five seven-day-and-eight-week-old Mongolian gerbils (*Meriones unguiculatus*), five seven-day-and-eight-week-old guinea pigs (*Cavia porcellus*), five seven-day-and-eight-week-old Southern multimammate mice (*Mastomys coucha*), five seven-day-old chickens (*Gallus gallus f. domestica*), and five adult budgerigars (*Melopsittacus undulatus*) were used for transmission studies. Three animals from each strain/species were used as a negative control. To prevent environmental contamination with *Cryptosporidium* spp., animals were housed in plastic cages/aviaries and supplied with a sterilized diet for appropriate host-species and sterilized water ad libitum. Seven-day-and-eight-week-old animals were each inoculated orally by stomach tube with 5000 purified viable oocysts suspended in 50 µL and 200 µL of distilled water, respectively. Faecal samples from all inoculated and control animals were collected daily for 20 days. All samples were screened for the presence of *Cryptosporidium* oocysts and specific DNA using microscopy (following ACMV staining) and PCR amplification of the *SSU* gene, respectively (methods described above). Animals were housed under conditions in accordance with Czech legislation (Act No 246/1992 Coll., on protection of animals against cruelty). Animal caretakers always wore sterile shoe covers, disposable coveralls, and disposable gloves when they entered the experimental room. Woodchip bedding and disposable protective clothing were removed from the experimental room and incinerated.

### 2.8. Clinical and Pathomorphological Examinations

All animals were sacrificed in accordance with Czech legislation (Act No 246/1992 Coll) at 20 days post-infection (DPI), and the complete examination of all gastrointestinal organs was conducted at necropsy. Tissue specimens from the oesophagus; stomach; duodenum; proximal, central and distal jejunum; ileum; caecum; colon; liver; spleen; kidney; bladder and lung were collected using different sterile dissection tools for each location and processed for histology [32], scanning electron microscopy (SEM) [33], and PCR amplification of the SSU gene. Histology sections (5 µm) were stained with hematoxylin and eosin (HE) and periodic acid-Schiff (PAS) and were examined at 100–400× magnification (Olympus IX70). Specimens for SEM were examined using a JEOL JSM-7401F-FE scanning electron microscope (Jeol, Tokyo, Japan).

### 2.9. Statistical Analysis

For the evaluation of difference in relative frequency in positivity between groups (country, age, gender), we used the two sample test for equality of proportions without continuity correction. Differences in oocyst sizes were tested using Hotelling’s multivariate version of the 2 sample *t*-test, package ICSNP: Tools for Multivariate Nonparametrics (Nordhausen et al. 2018) in R 4.1.0. [34]. The hypothesis tested was that two-dimensional mean vectors of measurement are the same in the two populations being compared.

## 3. Results

**Figure 2:**
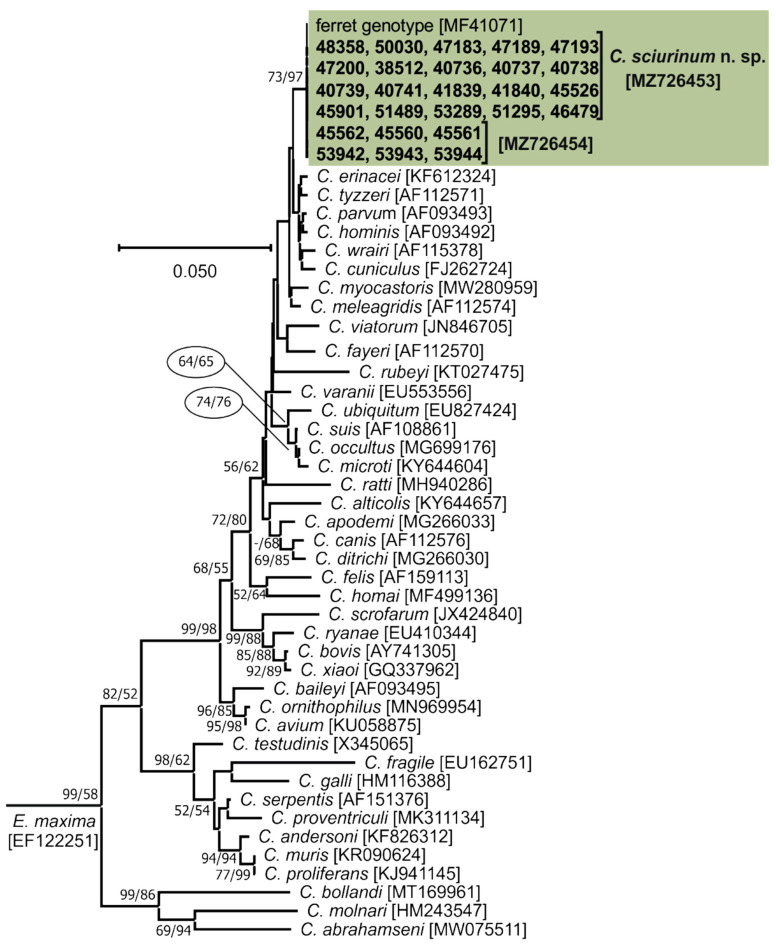
Evolutionary relationships of *Cryptosporidium* spp. at the small subunit rRNA (*SSU*) locus inferred by Maximum Likelihood (ML) and Neighbor-Joining (NJ) analyses using a General Time Reversible model with a gamma distribution: Percentage support (>50%) from 1000 pseudoreplicates from ML and NJ analysis, respectively, are indicated next to supported node. The ‘-’ indicates support value <50%. The tree is drawn to scale, with branch lengths in the same units as those of the evolutionary distances used to infer the phylogenetic tree. The analysis involved 70 nucleotide sequences and there were a total of 771 positions in the final dataset. The tree was rooted with the *SSU* sequence of *Eimeria maxima* [EF122251]. Sequences obtained in this study are identified by isolate number (e.g., 45901). The GenBank Accession number is in parenthesis. Isolates detected in this study are colour-coded.

**Figure 3:**
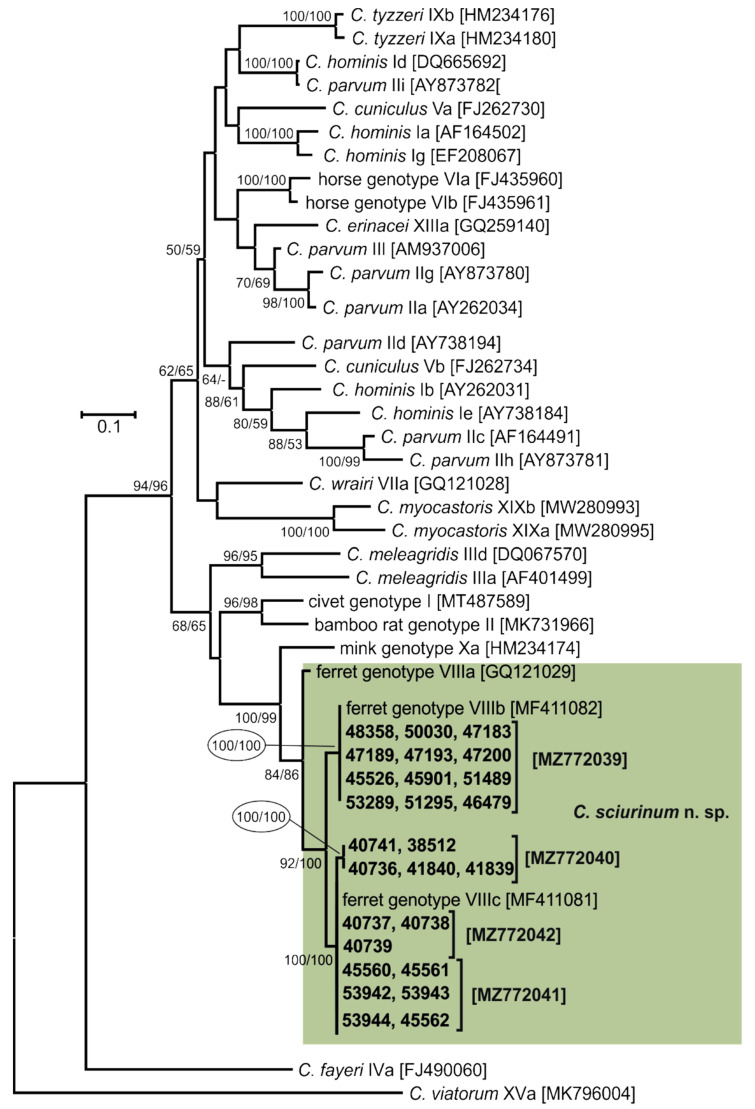
Evolutionary relationships of *Cryptosporidium* spp. at 60 kDa glycoprotein (*gp60*) locus inferred by Maximum Likelihood (ML) and Neighbor-Joining (NJ) analyses using a General Time Reversible model with a gamma distribution: Percentage support (>50%) from 1000 pseudoreplicates from ML and NJ analysis, respectively, are indicated next to supported node. The ‘-’ indicates support value <50%. The tree is drawn to scale, with branch lengths in the same units as those of the evolutionary distances used to infer the phylogenetic tree. The analysis involved 58 nucleotide sequences and there were a total of 1134 positions in the final dataset. The tree was rooted with the *gp60* sequence of *Cryptosporidium viatorum* [MK796004]. Sequences obtained in this study are identified by isolate number (e.g., 45901). The GenBank Accession number is in parenthesis. Isolates detected in this study are colour-coded.

*Cryptosporidium*-specific DNA was identified in 26 squirrels by nested PCR targeting the *SSU* gene (Table 1). Of these 26 squirrels, nine (34.6%) were microscopically positive for the presence of *Cryptosporidium* oocysts, with an infection intensity ranging from 18,000 to 72,000 OPG. Microscopically detectable infection was observed exclusively in juvenile squirrels. However, the occurrence of infection detected by PCR did not differ between juveniles (8.9%) and adults (11.8%; χ^2^ = 0.52991, *d.f.* = 1, *p*-value = 0.4666). Similarly, the occurrence of infection was not affected by host gender (χ^2^= 0.2966, *d.f.* = 1, *p*-value = 0.5860) or country of capture (χ^2^ = 0.0028, *d.f.* = 1, *p*-value = 0.9388; Table S1). Phylogenetic trees (ML, NJ) constructed from *SSU* sequences showed the presence of *Cryptosporidium* sp. ferret genotype in all samples (Figure 2). Out of the 26 *Cryptosporidium*-positive squirrels, 26, 26, 19, 16, 15, and 26 were genotyped by sequence analysis of the *SSU*, actin, *HSP70*, *TRAP-C1*, *COWP*, and *gp60* genes, respectively. For the remaining samples, either the PCR product was not amplified or the sequencing failed. For the actin, *HSP70*, *TRAP-C1*, and *COWP* genes, isolates of *Cryptosporidium* sp. ferret genotype in this study shared 100% identity with each other and 99.8–100% identity with previously reported sequences (Figures S1–S4). Sequences of the *gp60* gene were obtained from all 26 isolates and were identified as *Cryptosporidium* sp. ferret genotype subtype families VIIIb (*n* = 12) and VIIIc (*n* = 14; Figure 3).

**Figure 4:**
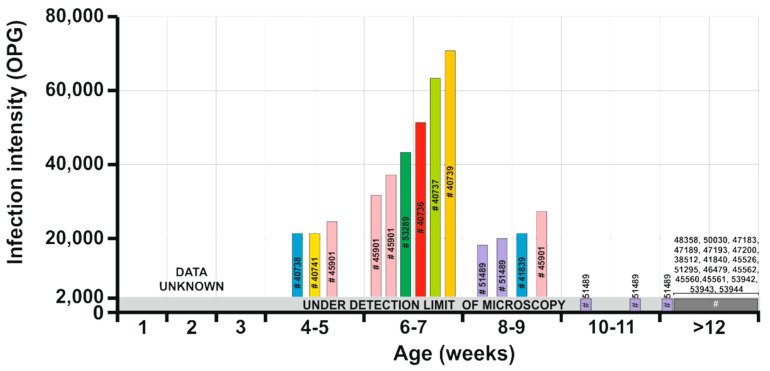
Infection intensity of *Cryptosporidium sciurinum* n. sp. in Eurasian red squirrels (*Sciurus vulgaris*) expressed as number of oocysts per gram of faeces (OPG). Data were collected from naturally infected Eurasian red squirrels. Animals aged 4–11 weeks old originated from rescue stations, and some of those animals were screened repeatedly (each animal is marked with a colour). The animal number (e.g., #45901) corresponds with the numbers in Table 1.

**Figure 5:**
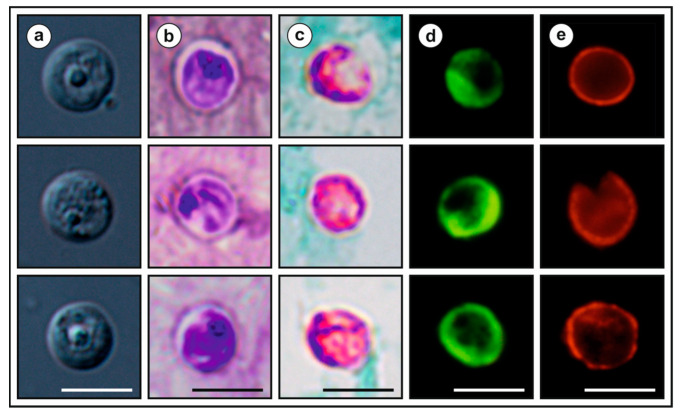
Oocysts of *Cryptosporidium sciurinum* n. sp. (**a**) under differential interference contrast microscopy, (**b**) stained by aniline-carbol-methyl violet staining, (**c**) stained by Ziehl–Nielsen staining, (**d**) labelled with anti-*Cryptosporidium* FITC-conjugated antibody, (**e**) labelled with anti-*Cryptosporidium* Cy3-conjugated antibody. Bar = 5 μm.

Microscopic, molecular, and histological examination of inoculated animals showed no evidence of infection in juvenile or adult SCID and BALB/c mice, Mongolian gerbils, guinea pigs, Southern multimammate mice, juvenile chickens, and adult budgerigars. For logistical reasons, we were unable to determine the infectivity of *Cryptosporidium* sp. ferret genotype for squirrels under experimental conditions. We therefore determined the infection intensity in squirrels of different ages kept at rescue stations. Nine of the eleven juvenile animals infected with *Cryptosporidium* sp. ferret genotype shed microscopically detectable oocysts (18,000–74,000 OPG). In contrast, oocyst numbers were below the detection limit (2000 OPG) in animals older than 10 weeks of age. The highest infection intensity was observed in animals aged 6–7 weeks (mean 49,666 OPG) compared to animals aged 4–5 weeks (mean 22,666 OPG) and 8–9 weeks (mean 21,000 OPG) (Figure 4). The consistency of the faeces and lack of faeces in the fur surrounding the rectum indicated an absence of diarrhoeal disease. Oocysts of *Cryptosporidium* sp. ferret genotype were spherical, with a thick, clear, and smooth oocyst wall. Oocysts from isolate 45901, which were measured in suspension after purification, measured 5.54 × 5.22 µm, with a length to width ratio of 1.07 (Figure 5a). There was no significant difference in oocyst size between isolate 45901 and other isolates (40793, 51489, and 53289; Table S2). Oocysts of *Cryptosporidium* sp. ferret genotype in the faecal smears stained by AVMC and ZN showed typical *Cryptosporidium* staining characteristics (Figure 5b,c) and cross-reacted with immunofluorescence reagents developed originally for *C. parvum* (Figure 5d,e). Oocysts that were dried and fixed onto slides for staining and labelling were smaller than those measured in suspension (data not shown).

Based on the data presented here, we propose *Cryptosporidium* sp. ferret genotype as a new species—*Cryptosporidium sciurinum* n. sp.—, whose description is presented below.

***Taxonomic summary***
**Family Cryptosporidiidae Léger, 1911**
**Genus *Cryptosporidium* Tyzzer, 1907**
***Cryptosporidiumsciurinum* n. sp.**
**Syn:** *Cryptosporidium parvum* ferret genotype ex black-footed ferret (*Mustela nigripes*) and *Cryptosporidium* sp. ferret genotype ex black-footed ferret (*Mustela nigripes*) of Xiao et al. [35].
**Type-host:***Sciurus vulgaris* Linnaeus, 1758 (Rodentia: Muridae) Eurasian red squirrel.
**Other natural hosts:** black-footed ferret (*Mustela nigripes*), Siberian chipmunk (*Tamias sibiricus*), Eastern chipmunk (*Tamias striatus*), budgerigar (*Melopsittacus undulatus*).
**Type-locality:** Třeboň, Czech Republic.
**Other localities:** České Budějovice, Brno, Praha, Vlašim, whole Czech Republic; Povážská Bystrica, Handlová, Gabčíkovo, Bratislava, Košice, whole Slovakia; Guangdong, China; Northern Italy; Georgia, USA.
**Type-material:** Faecal smear slides with oocysts stained by ACMV and ZN staining (nos. MV1-3/45901 and ZN1-2/45901) and gDNA isolated from faecal samples of a naturally infected Eurasian red squirrel (isolate 45901) are deposited at the Institute of Parasitology, Biology Centre of the Czech Academy of Sciences, Czech Republic.
**Site of infection:** unknown.
**Distribution:** As *Cryptosporidium* sp. ferret genotype ex *Sciurus vulgaris*: China, Italy [13,16,17]; as *Cryptosporidium* sp. ferret genotype or *Cryptosporidium parvum* ferret genotype ex *Mustela nigripes*: USA [35], as *Cryptosporidium* sp. ferret genotype ex *Tamias striatus*, *T. sibiricus* and *Melopsittacus undulatus*: China [13,36].
**Prepatent period:** unknown.
**Patent period:** At least 5 weeks in naturally infected *Sciurus vulgaris* (isolate 51489 in the present study).
**Representative DNA sequences**: Representative nucleotide sequences of *SSU* [MZ726453], actin [MZ772035], *HSP70* [MZ772047], *TRAP-C1* [MZ772037], *COWP* [MZ772045], and *gp60* [MZ772039–MZ772042] genes are deposited in the GenBank database.
**ZooBank registration:** To comply with the regulations set out in Article 8.5 of the amended 2012 version of the International Code of Zoological Nomenclature (ICZN) [37], details of the new species have been submitted to ZooBank. The Life Science Identifier (LSID) of the article is urn:lsid:zoobank.org:pub:83ABAD68-07C6-4E51-8234- A2AC4C0EE72C. The LSID for the new name *Cryptosporidium sciurinum* n. sp. is urn:lsid:zoobank.org:act:E13F2B9F-D9C6-4ED9-9EA8-B94AEB6189A2.
**Etymology:** The species name *sciurinum* is derived from the Latin noun sciurus, meaning squirrel.

**Description.**  Oocysts of *C. sciurinum* n. sp. (isolate 45901) are spherical, measuring 5.12–6.00 × 4.77–5.66 (5.54 ± 0.20 × 5.22 ± 0.18) with a length-to-width ratio of 1.00–1.26 (1.07 ± 0.05) (Figure 5). The oocyst wall is smooth and colourless (Figure 5a). The oocyst residuum is composed of numerous small granules and one spherical globule is clearly visible; a suture is not noticeable. Sporozoites are occasionally visible within oocysts. Morphology and morphometry of other developmental stages is unknown.

**Remark** **1.** Oocysts of *C. sciurinum* n. sp. are stained by ACMV and ZN staining methods, similar to other *Cryptosporidium* spp. (Figure 5b,c), and their oocyst wall cross reacts with immunofluorescence reagents developed originally for *C. parvum* (Figure 5d,e). Oocysts from naturally infected squirrels did not differ significantly in size (Table S2). Oocysts of *C. sciurinum* n. sp. are larger than those of *C. parvum* (T^2^ = 88.89, *df_1_* = 2, *df_2_* = 61.94, *p* < 0.001), *C. occultus* (T^2^ = 45.84, *df_1_* = 2, *df_2_* = 65.83, *p* < 0.001) and *C. ratti* (T^2^ = 88.22, *df_1_* = 2, *df_2_* = 24.75, *p* < 0.001), but these differences are not of practical significance for identification. *Cryptosporidium sciurinum* n. sp. can be differentiated genetically from other *Cryptosporidium* species based on sequences of *SSU*, actin, *HSP70*, *TRAP-C1*, *COWP*, and *gp60* genes. At *gp60* locus, *C. sciurinum* n. sp. is known to form three subtype families VIIIa, VIIIb, and VIIIc.

## 4. Discussion

Molecular studies conducted on three continents (North America, Europe, and Asia) have shown that tree squirrels host a variety of *Cryptosporidium* spp. [9,10,11,12,13,16,17]. While wild North American and Asian tree squirrels can be infected by a large number of species and genotypes of the genus *Cryptosporidium* [9,10,11,12,13], results from this and other studies suggest that wild Eurasian red squirrels are predominantly parasitized by *C. sciurinum* n. sp. [13,16,17]. The only exceptions are two cases of *Cryptosporidium* sp. chipmunk genotype I, which naturally infects grey squirrels, and two cases of *C. parvum* reported in Eurasian red squirrels in Italy [13,16,17]. In contrast to wild animals, Eurasian red squirrels kept as caged pets in China were found to be infected with *C. ratti* and *Cryptosporidium* sp. chipmunk genotype III, which are specific for other hosts [13]. In the present study, 10.1% (26/258) of Eurasian red squirrels were infected with *C. sciurinum* n. sp. Previous studies of *C. sciurinum* n. sp. in Eurasian red squirrels have reported prevalences of 10.6% (13/123) and 21.5% (15/70) in Italy and 26.3% in China [13,16,17]. The prevalence range of *Cryptosporidium* is similar with that found in other wild rodents, such as 14% in *Apodemus* spp. in Europe, 27% in *Apodemus speciosus* in Japan, 16% in brown rats (*Rattus norvegicus*) in Czech Republic, 12% in muskrats (*Ondatra zibethicus*) in USA, 30% in Chinese bamboo rats (*Rhizomys sinensis*) in China, or 7–14% in voles in Europe [38,39,40,41,42]. Consistent with most reports describing natural infections with *Cryptosporidium* spp. in wild rodents [16,17,38,42,43,44], Eurasian red squirrels infected with *C. sciurinum* n. sp. shed low numbers of oocysts, often below the detection limit of microscopy. A characteristic of *C. sciurinum* n. sp. infection in Eurasian red squirrels is that mostly juveniles shed oocysts at levels that are detectable by microscopy [16,17]. This age-dependent variation in *Cryptosporidium* spp. infection intensity has been observed previously in several studies [44,45,46,47,48,49]. Juveniles are probably more susceptible to infection because of their naive and immature immune system, which permits a higher intensity of infection. The prepatent period of *C. sciurinum* n. sp. is unknown. From the results we have collected, it is clear that pups become infected at a very early age (as early as 4 weeks), and that the infection persists for more than a month. It is possible that asymptomatically infected adults are a source of infection for their young, but this would need to be investigated experimentally. The pathogenicity of *C. sciurinum* n. sp. is unknown; however, based on the observations of naturally infected squirrels in rescue stations, this *Cryptosporidium* species appears to have low pathogenicity for infected individuals. This finding is consistent with previously published results [16,17].

*Cryptosporidium sciurinum* n. sp. was firstly identified in three naturally infected ferrets (reported as *C. parvum* ferret genotype (AF112572)) [35]. Since then, this species has been found in five captive chipmunks (*T. sibiricus* and *T. striatus*), one budgerigar (*M. undulatus*), and dozens of wild and captive Eurasian red squirrels [13,15,16,17,35,36], suggesting that Eurasian red squirrels are a natural host of this parasite. This is supported by the finding in the present study that *C. sciurinum* n. sp. is not infective for SCID and BALB/c mice, Mongolian gerbils, guinea pigs, Southern multimammate mice, chickens, and budgerigars. The solitary infection reported in a budgerigar [36] could be the result of environmental contamination and the passage of oocysts or specific DNA through the digestive tract of a non-specific host, as has been described previously [50,51,52,53].

Oocysts of *C. sciurinum* n. sp. measured 5.54 × 5.22 μm, which is similar in size to a previously published isolate of *C. sciurinum* n. sp. (5.5 × 5.2 μm). *Cryptosporidium sciurinum* n. sp. is larger than *C. parvum* (5.2 × 4.9 μm) and *C. ratti* (4.9 × 4.6 μm), and is smaller than *Cryptosporidium* sp. chipmunk genotype I (5.8 × 5.4 μm), a species previously reported in Eurasian red squirrels [16,40]. However, the difference in size is not practically useful for distinguishing species by routine microscopy.

Although a description of oocyst morphology is a requirement for species description, molecular characterisation is necessary for species identification. Several previous studies have shown the presence of divergent copies of *SSU* gene within a *Cryptosporidium* species. Stenger et al. [54] found highly divergent SSU genotypes in *Cryptosporidium* sp. chipmunk genotype II. The co-occurrence of the genotypes in a host and the homogeneity of actin and *HSP70* sequences supported the conclusion that the divergent types are paralogs in a single *Cryptosporidium* lineage. Similarly, divergent types of *SSU* have been reported within, e.g., *C. andersoni*, *C. apodemi*, *C. ditrichi*, *C. parvum*, *C. ubiquitum*, *Cryptosporidium* sp. apodemus genotype I and II, or *Cryptosporidium* rat genotype II and III [15,42,43,55,56]. As inferring the evolutionary relationships of *Cryptosporidium* spp. using *SSU* sequences alone can lead to erroneous conclusions [54,57,58], we included other polymorphic loci for our analyses. Although some previous studies have shown polymorphism in actin, *HSP70*, and *TRAP-C1* genes in *Cryptosporidium* spp. [42,43,56], studies from our group and others have failed to find variants of these genes in *C. sciurinum* n. sp. [17,24,25]. All isolates of *C. sciurinum* n. sp. from the present study shared 100% identity at the COWP locus and differed from a sequence obtained from ferret (*Mustela putorius furo*) in Japan (AB469366) by a synonymous SNP (C/T) at position 387. A similar synonymous SNP at the COWP locus was observed in geographically distinct isolates of *C. tyzzeri* [59].

Phylogenetic analyses of *C. sciurinum* n. sp. at *SSU*, actin, *HSP70*, *TRAP-C1*, *COWP*, and *gp60* loci confirmed its status as a separate species from valid *Cryptosporidium* species. At all loci, *C. sciurinum* n. sp. formed a separate clade within the group of intestinal *Cryptosporidium* spp. that includes *C. meleagridis*, *C. parvum*, *C. hominis*, *C. wrairi*, and *C. tyzzeri*. At actin, *HSP70*, *TRAP-C1*, and *COWP* loci, the pairwise distances between *C. sciurinum* n. sp. and *C. meleagridis* (0.026, 0.036, 0.050, and 0.024, respectively) are similar to those between *C. andersoni* and *C. muris* (0.037, 0.018, 0.041, and 0.018, respectively) and *C. hominis* and *C. parvum* (0.018, 0.013, 0.018, and 0.016, respectively).

At the *gp60* locus, only subtype families VIIIb and VIIIc were detected in the present study. Both subtypes have been found exclusively in Europe. In contrast, subtype VIIIa was found exclusively in China [13]. Other studies have found that *gp60* subtype families differ in their geographic distribution. For example, *C. tyzzeri* subtype IXa has been detected only in house mice (*Mus musculus musculus*) inhabiting Eastern Europe, China, and Kuwait, while subtype IXb is found (*Mus musculus domesticus*) in Western Europe, USA, and New Zealand [59]. A similar difference in distribution has been reported for *C. parvum* and *C. ubiquitum gp60* subtypes [57,60]. It should be noted that gp60 subtype family VIIId was incorrectly reported in Eurasian red squirrels in Italy (the sequences actually belonged to *C. parvum* subtype IIa) and it should no longer be used for *C. sciurinum* n. sp. [17].

## 5. Conclusions

In summary, the present study confirms that *Cryptosporidium* sp. ferret genotype is genetically distinct from all currently accepted species of the genus *Cryptosporidium*, and its specificity for tree squirrels under natural conditions supports its description as a new species, which we propose be named *Cryptosporidium* *sciurinum* n. sp.

## Figures and Tables

**Table 1:** The occurrence and genetic diversity of *Cryptosporidium* spp. in Eurasian red squirrels (*Sciurus vulgaris*) trapped in the wild (W) and screened at rescue stations (RS) in the Czech Republic and Slovakia. Detected isolates were genotyped by sequence analysis of the small subunit ribosomal rRNA (*SSU*), actin, 70 kDa heat-shock protein (*HSP70*), thrombospondin-related adhesive protein of *Cryptosporidium*-1 (*TRAP-C1*), *Cryptosporidium* oocyst wall protein (*COWP*), and 60 kDa glycoprotein (*gp60*) gene fragments. Oocysts were quantified by microscopy and were reported per gram of faeces (OPG).

Country	Locality	Type	No. of Screened/No. of Positive	ID of Positive Animal	Microscopical Positivity	Sex/Age	Genotyping at the Gene Loci					
							*SSU*	Actin	*HSP70*	*COWP*	*TRAP-C1*	*gp60*
Czech Republic	(1)	W	2/0	-	-	-	-	-	-	-	-	-
	(2)	W	1/0	-	-	-	-	-	-	-	-	-
	(3)	W	5/2	48358	No	F/A	*C. sciurinum*	*C. sciurinum*	*C. sciurinum*	*C. sciurinum*	*C. sciurinum*	VIIIbA11G1R1
				50030	No	M/A	*C. sciurinum*	*C. sciurinum*	-	*C. sciurinum*	-	VIIIbA11G1R1
	(4)	W	2/0	-	-	-	-	-	-	-	-	-
	(5)	W	15/4	47183	No	M/J	*C. sciurinum*	*C. sciurinum*	*C. sciurinum*	-	*C. sciurinum*	VIIIbA11G1R1
				47189	No	F/A	*C. sciurinum*	*C. sciurinum*	-	ferret genotype	-	VIIIbA11G1R1
				47193	No	M/A	*C. sciurinum*	*C. sciurinum*	*C. sciurinum*	-	*C. sciurinum*	VIIIbA11G1R1
				47200	No	F/J	*C. sciurinum*	*C. sciurinum*	*C. sciurinum*	*C. sciurinum*	-	VIIIbA11G1R1
	(6)	W	1/0	-	-	-	-	-	-	-	-	-
	(7)	W	2/0	-	-	-	-	-	-	-	-	-
	(8)	W	4/0	-	-	-	-	-	-	-	-	-
	(9)	W	3/0	-	-	-	-	-	-	-	-	-
	(10)	W	3/0	-	-	-	-	-	-	-	-	-
	(11)	W	7/1	38512	No	F/A	*C. sciurinum*	*C. sciurinum*	*C. sciurinum*	-	-	VIIIcA10G2R1
	(12)	W	3/0	-	-	-	-	-	-	-	-	-
	(13)	W	6/0	-	-	-	-	-	-	-	-	-
	(14)	W	5/0	-	-	-	-	-	-	-	-	-
	(15)	W	5/0	-	-	-	-	-	-	-	-	-
	(16)	W	3/0	-	-	-	-	-	-	-	-	-
	(17)	W	4/0	-	-	-	-	-	-	-	-	-
	(18)	W	5/0	-	-	-	-	-	-	-	-	-
	(19) J	RC	14/0	-	-	-	-	-	-	-	-	-
	(20)	RC	4/0	-	-	-	-	-	-	-	-	-
	(21)	RC	37/5	40736	Yes	F/J	*C. sciurinum*	*C. sciurinum*	*C. sciurinum*	*C. sciurinum*	-	VIIIcA10G2R1
				40737	Yes	M/J	*C. sciurinum*	*C. sciurinum*	*C. sciurinum*	*C. sciurinum*	*C. sciurinum*	VIIIcA10G2R1
				40738	Yes	F/J	*C. sciurinum*	*C. sciurinum*	*C. sciurinum*	*C. sciurinum*	-	VIIIcA10G2R1
				40739	Yes	M/J	*C. sciurinum*	*C. sciurinum*	-	*C. sciurinum*	*C. sciurinum*	VIIIcA10G2R1
				40741	Yes	M/J	*C. sciurinum*	*C. sciurinum*	*C. sciurinum*	*C. sciurinum*	*C. sciurinum*	VIIIcA10G2R1
	(22 )	RC	23/2	41839	Yes	F/J	*C. sciurinum*	*C. sciurinum*	*C. sciurinum*	*C. sciurinum*	*C. sciurinum*	VIIIcA10G2R1
				41840	No	M/A	*C. sciurinum*	*C. sciurinum*	-	-	*C. sciurinum*	VIIIcA10G2R1
	(23)	RC	21/5	45526	No	M/A	*C. sciurinum*	*C. sciurinum*	*C. sciurinum*	-	*C. sciurinum*	VIIIbA11G1R1
				45901	Yes	F/J	*C. sciurinum*	*C. sciurinum*	*C. sciurinum*	*C. sciurinum*	*C. sciurinum*	VIIIbA11G1R1
				51489	Yes	M/J	*C. sciurinum*	*C. sciurinum*	*C. sciurinum*	*C. sciurinum*	-	VIIIbA11G1R1
				53289	Yes	F/J	*C. sciurinum*	*C. sciurinum*	*C. sciurinum*	*C. sciurinum*	*C. sciurinum*	VIIIbA11G1R1
				51295	No	M/A	*C. sciurinum*	*C. sciurinum*	-	-	*C. sciurinum*	VIIIbA11G1R1
	(24)	RC	15/1	46479	No	F/A	*C. sciurinum*	*C. sciurinum*	*C. sciurinum*	-	-	VIIIbA11G1R1
	(25)	RC	15/0	-	-	-	-	-	-	-	-	-
Slovakia	(26)	W	8/0	-	-	-	-	-	-	-	-	-
	(27)	W	2/0	-	-	-	-	-	-	-	-	-
	(28)	W	5/1	45562	No	M/A	*C. sciurinum*	*C. sciurinum*	-	*C. sciurinum*	*C. sciurinum*	VIIIcA10G2R1
	(29)	W	2/0	-	-	-	-	-	-	-	-	-
	(30)	W	2/0	-	-	-	-	-	-	-	-	-
	(31)	W	5/1	45560	No	F/J	*C. sciurinum*	*C. sciurinum*	*C. sciurinum*	-	*C. sciurinum*	VIIIcA10G2R1
	(32)	W	4/0	-	-	-	-	-	-	-	-	-
	(33)	W	2/0	-	-	-	-	-	-	-	-	-
	(34)	W	6/0	-	-	-	-	-	-	-	-	-
	(35)	W	1/0	-	-	-	-	-	-	-	-	-
	(36)	W	4/0	-	-	-	-	-	-	-	-	-
	(37)	W	1/1	45561	No	F/A	*C. sciurinum*	*C. sciurinum*	*C. sciurinum*	*C. sciurinum*	-	VIIIcA10G2R1
	(38)	W	1/0	-	-	-	-	-	-	-	-	-
	(39)	W	7/2	53942	No	F/A	*C. sciurinum*	*C. sciurinum*	-	-	*C. sciurinum*	VIIIcA10G2R1
				53943	No	M/A	*C. sciurinum*	*C. sciurinum*	*C. sciurinum*	*C. sciurinum*	-	VIIIcA10G2R1
	(40)	W	8/1	53944	No	M/A	*C. sciurinum*	*C. sciurinum*	*C. sciurinum*	-	-	VIIIcA10G2R1

## Data Availability

Data is contained within the article or supplementary material.

## Supplementary Materials

The following are available online at https://www.mdpi.com/article/10.3390/microorganisms9102050/s1: **Figure S1.** Evolutionary relationships of *Cryptosporidium* spp. at the actin locus inferred by Maximum Likelihood (ML) and Neighbor-Joining (NJ) analyses using the Tamura-3-parameter model with a gamma distribution: Percentage support (>50%) from 1000 pseudoreplicates from ML and NJ analysis, respectively, are indicated next to supported node. The ‘-’ indicates support value <50%. The tree is drawn to scale, with branch lengths in the same units as those of the evolutionary distances used to infer the phylogenetic tree. The analysis involved 69 nucleotide sequences and there were a total of 727 positions in the final dataset. The tree was rooted with the actin sequence of *Plasmodium* sp. [KF159609]. Sequences obtained in this study are identified by isolate number (e.g., 45901). The GenBank Accession number is in parenthesis. Isolates detected in this study are colour-coded. **Figure S2.** Evolutionary relationships of *Cryptosporidium* spp. at 70 kDa heat-shock protein (*HSP70*) locus inferred by Maximum Likelihood (ML) and Neighbor-Joining (NJ) analyses using a General Time Reversible model with a gamma distribution: Percentage support (>50%) from 1000 pseudoreplicates from ML and NJ analysis, respectively, are indicated next to supported node. The ‘-’ indicates support value <50%. The tree is drawn to scale, with branch lengths in the same units as those of the evolutionary distances used to infer the phylogenetic tree. The analysis involved 69 nucleotide sequences and there were a total of 1728 positions in the final dataset. The tree was rooted with the *HSP70* sequence of *Eimeria maxima* [Z46964]. Sequences obtained in this study are identified by isolate number (e.g., 45901). The GenBank Accession number is in parenthesis. Isolates detected in this study are colour-coded. **Figure S3.** Evolutionary relationships of *Cryptosporidium* spp. at thrombospondin-related adhesive protein of *Cryptosporidium*-1 (*TRAP-C1*) locus inferred by Maximum Likelihood (ML) and Neighbor-Joining (NJ) analyses using a General Time Reversible model with a gamma distribution: Percentage support (>50%) from 1000 pseudoreplicates from ML and NJ analysis, respectively, are indicated next to supported node. The ‘-’ indicates support value <50%. The tree is drawn to scale, with branch lengths in the same units as those of the evolutionary distances used to infer the phylogenetic tree. The analysis involved 29 nucleotide sequences and there were a total of 470 positions in the final dataset. The tree was rooted with the *TRAP-C1* sequence of gastric *Cryptosporidium* spp. Sequences obtained in this study are identified by isolate number (e.g., 45901). The GenBank Accession number is in parenthesis. Isolates detected in this study are colour-coded. **Figure S4.** Evolutionary relationships of *Cryptosporidium* spp. at *Cryptosporidium* oocyst wall protein (*COWP*) locus inferred by Maximum Likelihood (ML) and Neighbor-Joining (NJ) analyses using a General Time Reversible model with a gamma distribution: Percentage support (>50%) from 1000 pseudoreplicates from ML and NJ analysis, respectively, are indicated next to supported node. The ‘-’ indicates support value <50%. The tree is drawn to scale, with branch lengths in the same units as those of the evolutionary distances used to infer the phylogenetic tree. The analysis involved 41 nucleotide sequences and there were a total of 477 positions in the final dataset. The tree was rooted with the *COWP* sequence of gastric *Cryptosporidium* spp. Sequences obtained in this study are identified by isolate number (e.g., 45901). The GenBank Accession number is in parenthesis. Isolates detected in this study are colour-coded. **Table S1.** Occurrence of *Cryptosporidium sciurinum* n. sp. in faeces of Eurasian red squirrels (*Sciurus vulgaris*) based on microscopic and molecular examination. **Table S2.** Size of *Cryptosporidium sciurinum* n. sp. oocysts obtained from naturally infected Eurasian red squirrels (*Sciurus vulgaris*).
