# Supplementary material for: Cryptosporidium sciurinum n. sp. (Apicomplexa: Cryptosporidiidae) in Eurasian Red Squirrels (Sciurus vulgaris)
Source: Microorganisms. 2021 Sep 28;9(10):2050. doi: 10.3390/microorganisms9102050 (PMC8537388; doi:10.3390/microorganisms9102050)
Supplement: Supplementary file 1 [file microorganisms-09-02050-s001.zip › microorganisms-1368313-supplementary.pdf]

Appendix A. Supplementary data

The following is the supplementary data related to the article ……

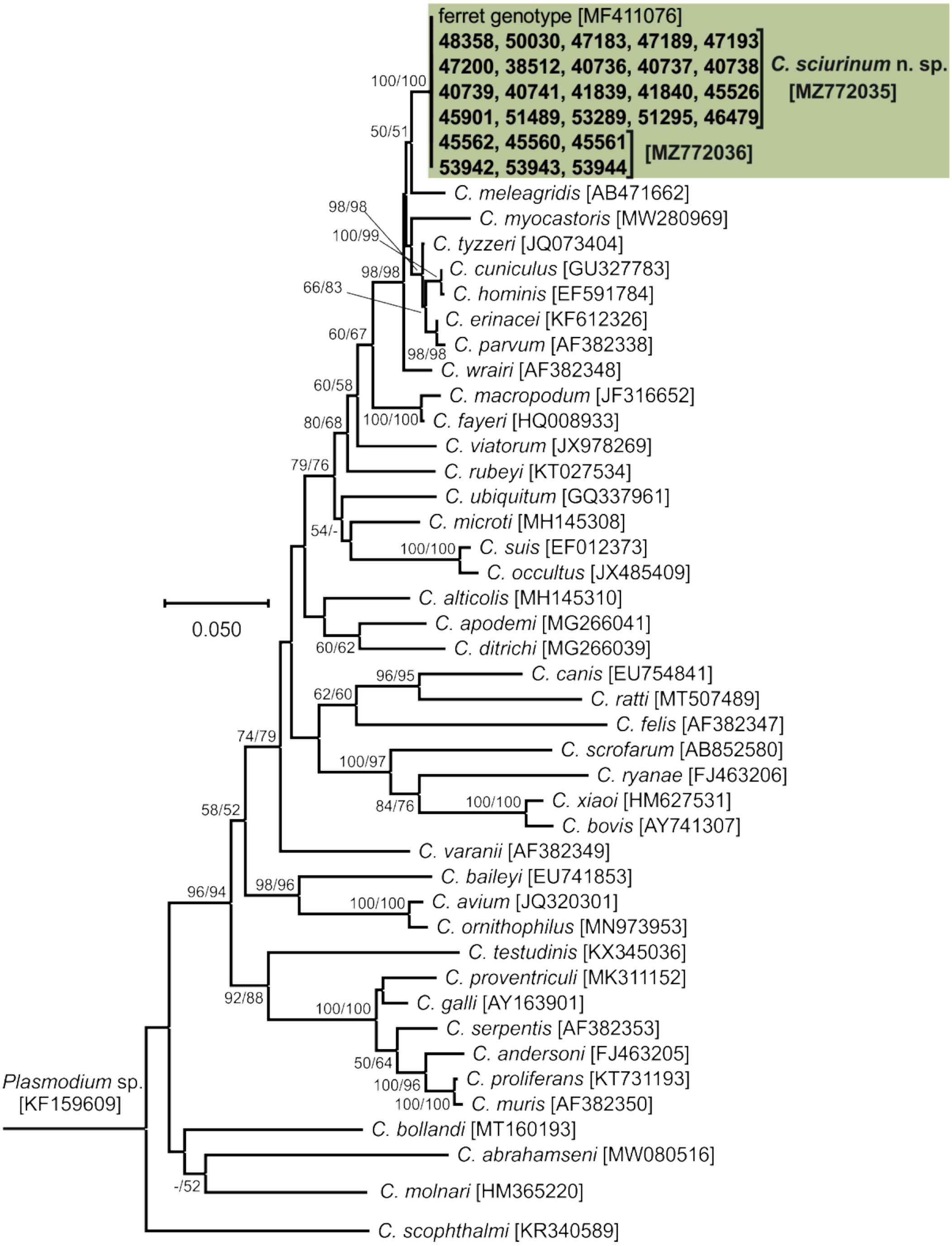

Figure S1. Evolutionary relationships of *Cryptosporidium* spp. at the actin locus inferred by Maximum Likelihood (ML) and Neighbor-Joining (NJ) analyses using the Tamura-3-parameter model with a gamma distribution. Percentage support (>50%) from 1000 pseudoreplicates from ML and NJ analysis, respectively, are indicated next to supported node. The '-' indicates support value <50%. The tree is drawn to scale, with branch lengths in the same units as those of the evolutionary distances used to infer the phylogenetic tree. The analysis involved 69 nucleotide sequences and there were a total of 727 positions in the final dataset. The tree was rooted with the actin sequence of *Plasmodium* sp. [KF159609]. Sequences obtained in this study are identified by isolate number (e.g. 45901). The GenBank Accession number is in parenthesis. Isolates detected in this study are colour-coded.

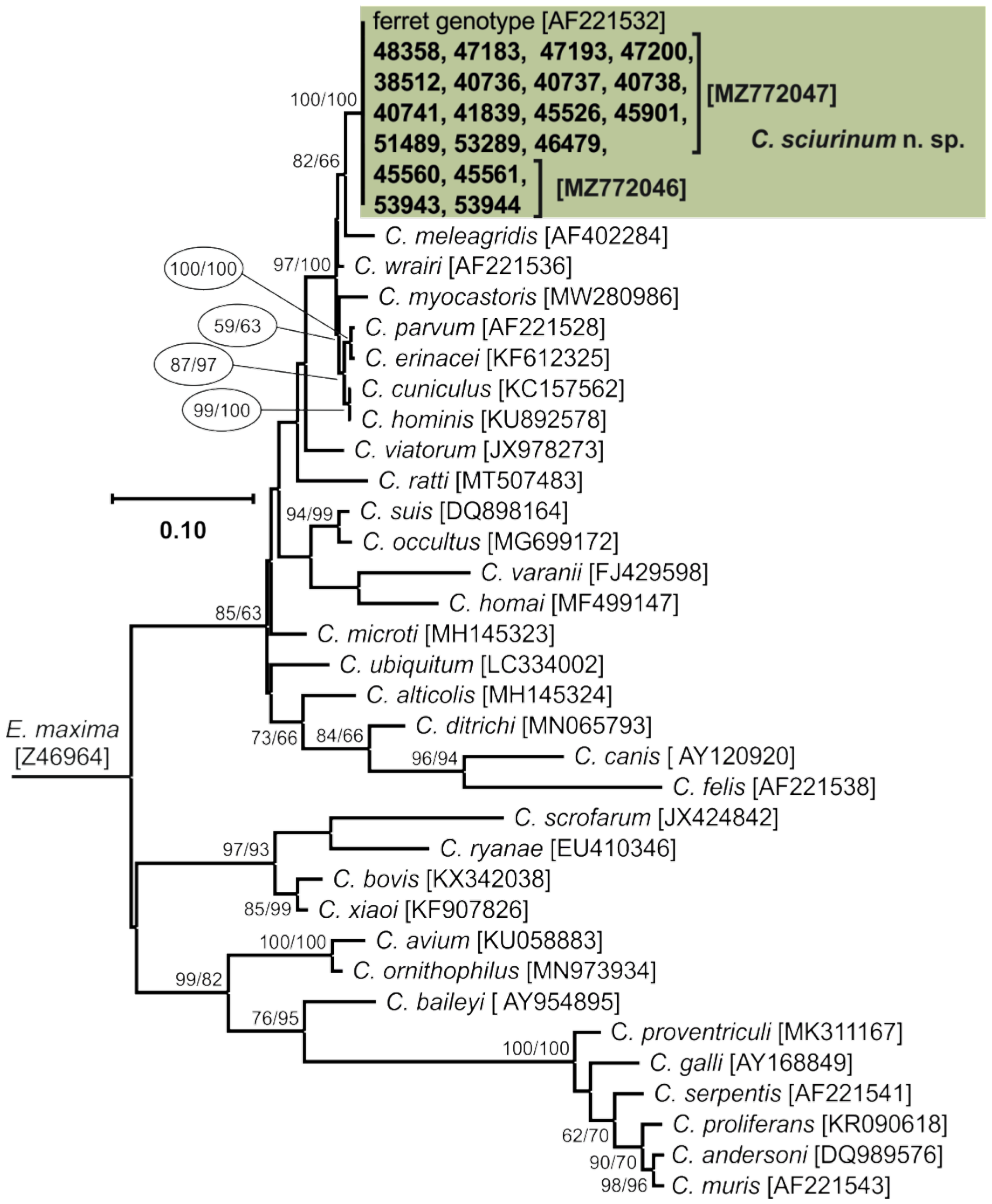

Figure S2. Evolutionary relationships of *Cryptosporidium* spp. at 70 kDa heat-shock protein (*HSP70*) locus inferred by Maximum Likelihood (ML) and Neighbor-Joining (NJ) analyses using a General Time Reversible model with a gamma distribution. Percentage support (>50%) from 1000 pseudoreplicates from ML and NJ analysis, respectively, are indicated next to supported node. The '-' indicates support value <50%. The tree is drawn to scale, with branch lengths in the same units as those of the evolutionary distances used to infer the phylogenetic tree. The analysis involved 69 nucleotide sequences and there were a total of 1728 positions in the final dataset. The tree was rooted with the *HSP70* sequence of *Eimeria maxima* [Z46964]. Sequences obtained in this study are identified by isolate number (e.g. 45901). The GenBank Accession number is in parenthesis. Isolates detected in this study are colour-coded.

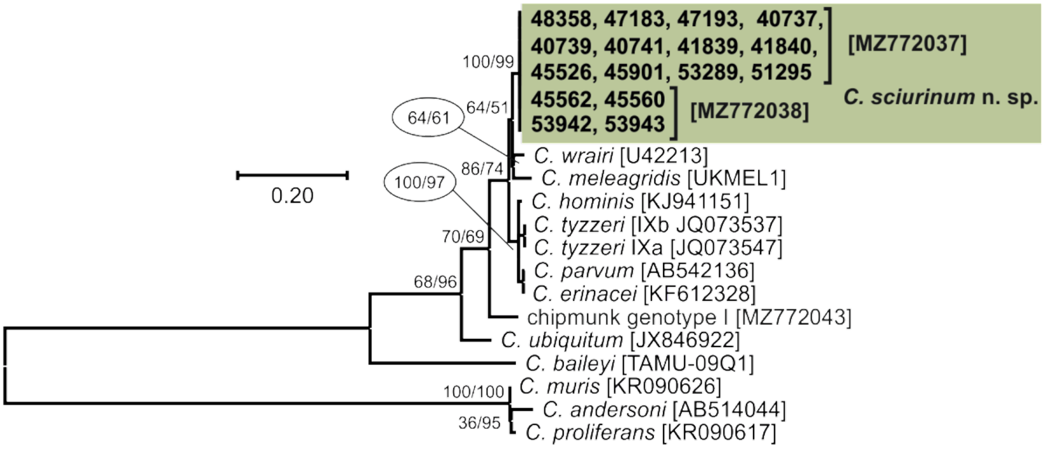

Figure S3. Evolutionary relationships of *Cryptosporidium* spp. at thrombospondin-related adhesive protein of *Cryptosporidium*-1 (*TRAP-C1*) locus inferred by Maximum Likelihood (ML) and Neighbor-Joining (NJ) analyses using a General Time Reversible model with a gamma distribution. Percentage support (>50%) from 1000 pseudoreplicates from ML and NJ analysis, respectively, are indicated next to supported node. The '-' indicates support value <50%. The tree is drawn to scale, with branch lengths in the same units as those of the evolutionary distances used to infer the phylogenetic tree. The analysis involved 29 nucleotide sequences and there were a total of 470 positions in the final dataset. The tree was rooted with the *TRAP-C1* sequence of gastric *Cryptosporidium* spp. Sequences obtained in this study are identified by isolate number (e.g. 45901). The GenBank Accession number is in parenthesis. Isolates detected in this study are colour-coded.

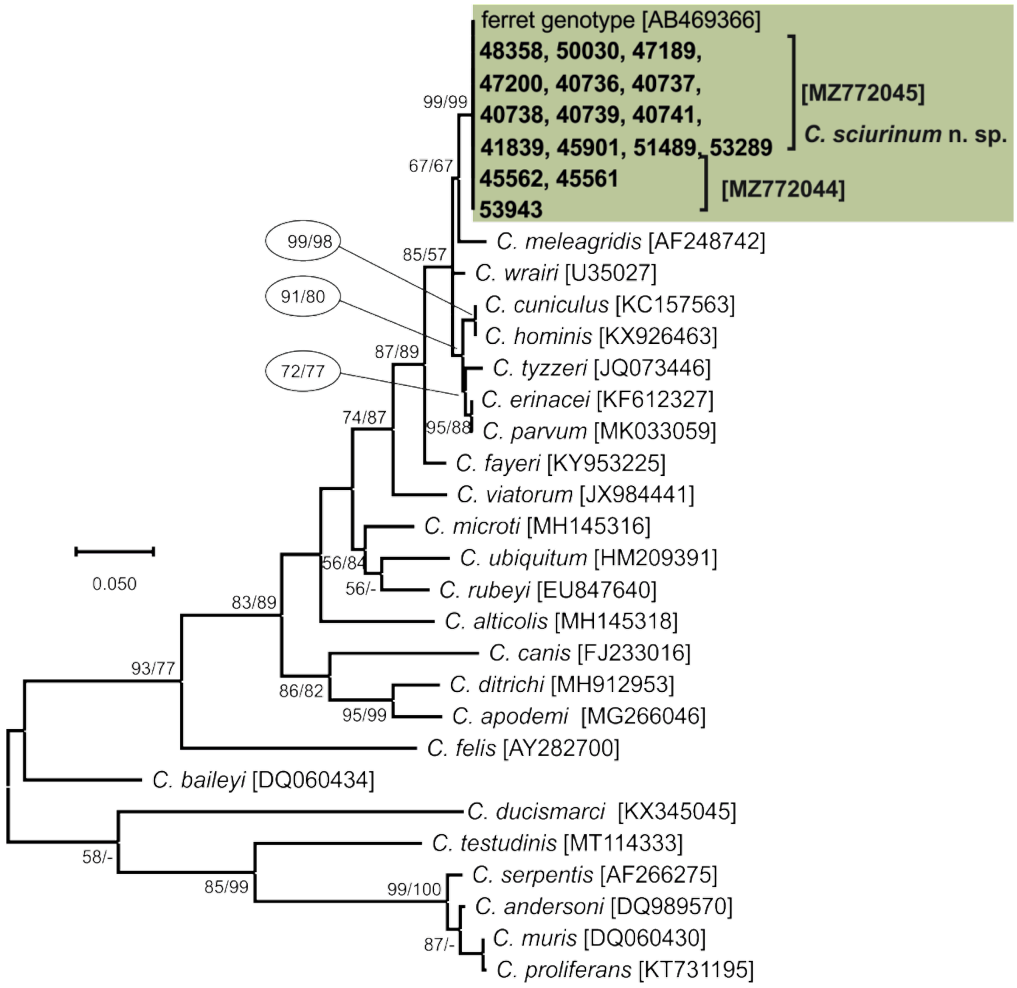

Figure S4. Evolutionary relationships of *Cryptosporidium* spp. at thrombospondin-related adhesive protein of *Cryptosporidium*-1 (*TRAP-C1*) locus inferred by Maximum Likelihood (ML) and Neighbor-Joining (NJ) analyses using a General Time Reversible model with a gamma distribution. Percentage support (>50%) from 1000 pseudoreplicates from ML and NJ analysis, respectively, are indicated next to supported node. The '-' indicates support value <50%. The tree is drawn to scale, with branch lengths in the same units as those of the evolutionary distances used to infer the phylogenetic tree. The analysis involved 29 nucleotide sequences and there were a total of 470 positions in the final dataset. The tree was rooted with the *TRAP-C1* sequence of gastric *Cryptosporidium* spp. Sequences obtained in this study are identified by isolate number (e.g. 45901). The GenBank Accession number is in parenthesis. Isolates detected in this study are colour-coded.

Table S1. Occurrence of *Cryptosporidium sciurinum* n. sp. in faeces of Eurasian red squirrels (*Sciurus vulgaris*) based on microscopic and molecular examination.

Country	Sex	Age	No. of samples	Microscopical positivity	PCR positivity
**Czech Republic**	Female	Adult	47	0	4
		Juvenile	43	5	6
	Male	Adult	61	0	5
		Juvenile	49	4	5
**Slovakia**	Female	Adult	21	0	2
		Juvenile	5	0	1
	Male	Adult	27	0	3
		Juvenile	5	0	0

Table S2. Size of *Cryptosporidium sciurinum* n. sp. oocysts obtained from naturally infected Eurasian red squirrels (*Sciurus vulgaris*).

Isolate no.	Length (μm)<br>Range (Mean ± SD)	Width (μm)<br>Range (Mean ± SD)	Length/width ratio<br>Range (Mean ± SD)
45901	5.12–6.00 (5.54 ± 0.20)	4.77–5.66 (5.22 ± 0.18)	1.00–1.26 (1.07 ± 0.05)
40793	5.21–5.90 (5.49 ± 0.22)	4.69–5.63 (5.15 ± 0.21)	1.05–1.27 (1.08 ± 0.06)
51489	5.23–6.15 (5.52 ± 0.26)	4.81–5.62 (5.18 ± 0.17)	1.10–1.26 (1.08 ± 0.08)
53289	5.19–5.96 (5.54 ± 0.18)	4.79–5.71 (5.26 ± 0.25)	1.07–1.29 (1.07 ± 0.05)

Note: Length and width of 50 oocysts from each isolate were measured under differential interference contrast at 1000× magnification, and out of these the length-to-width ratio of each oocyst was used to calculate.
